# Developing the Pieta House Suicide Intervention Model: a quasi-experimental, repeated measures design

**DOI:** 10.1186/s40359-015-0071-6

**Published:** 2015-05-02

**Authors:** Paul WG Surgenor, Joan Freeman, Cindy O’Connor

**Affiliations:** Pieta House, 6 Main Street Lucan, Co. Dublin, Ireland

**Keywords:** Suicide, Crisis, Intervention, Therapy, Model, Pieta House

## Abstract

**Background:**

While most crisis intervention models adhere to a generalised theoretical framework, the lack of clarity around how these should be enacted has resulted in a proliferation of models, most of which have little to no empirical support. The primary aim of this research was to propose a suicide intervention model that would resolve the client’s suicidal crisis by decreasing their suicidal ideation and improve their outlook through enhancing a range of protective factors. The secondary aim was to assess the impact of this model on negative and positive outlook.

**Methods:**

A quasi-experimental, pre-test post-test repeated measures design was employed. A questionnaire assessing self-esteem, depression, and positive and negative suicidal ideation was administered to the same participants pre- and post- therapy facilitating paired responses.

**Results:**

Multiple analysis of variance and paired-samples t-tests were conducted to establish whether therapy using the PH-SIM had a significant effect on the clients’ negative and positive outlook. Analyses revealed a statistically significant effect of therapy for depression, negative suicidal ideation, self-esteem, and positive suicidal ideation. Negative outlook was significantly lower after therapy and positive outlook significantly higher.

**Conclusions:**

The decreased negative outlook and increased positive outlook following therapy provide some support for the proposed model in fulfilling its role, though additional research is required to establish the precise role of the intervention model in achieving this.

## Background

### Introduction

A suicidal crisis requires an immediate and reliable intervention treatment. Unfortunately the dearth of intervention studies (Huisman et al. 2010) has limited our knowledge and options for empirically tested therapy models (Linehan 2008). The aim of this paper is to propose an intervention model that will support individuals through their immediate and future suicidal crises, and then to ascertain the impact of engaging in this therapy model on levels of negative and positive suicidal outlook.

### Definitions

Suicide research often suffers from definitional ambiguity (Linehan 1997). Consequently, this research adheres to the definitions of suicide (“a conscious or deliberate act that ends one’s life when an individual is attempting to solve a problem that is perceived as unsolvable by any other means”) and suicidal behaviour (“the spectrum of activities related to suicide including suicidal thinking, self-harming behaviours not aimed at causing death and suicide attempts”) used in the Irish National Strategy for Action on Suicide Prevention (National Office for Suicide Prevention 2005).

The proposed model is to assist clients in a state of crisis, defined by Roberts as “a period of psychological disequilibrium, experienced as a hazardous event or situation that constitutes a significant problem that cannot be remedied by using familiar coping strategies” (Roberts 2000) (p7). Consequently, the focus of this study is suicide intervention rather than prevention, with the former aiming to alter the course of existing ideation while the latter attempts to reduce the likelihood of risk or onset (Office of the Surgeon General (US) and National Action Alliance for Suicide Prevention (US) (2012)).

### Suicide intervention at Pieta House

Pieta House is an Irish suicide intervention charity that provides free counselling for those affected by suicide or deliberate self-harm. Therapy is founded on Shneidman’s (1985) assertion that while part of the individual wants to die another part wants to live and, if navigated successfully, suicidal crises need not be fatal. The therapy model necessitated by Pieta House must therefore provide an effective and immediate intervention that can be shown to redress the client’s wish to die and strengthen their will to live, a focus often neglected in intervention models (Ramsay 2004). Furthermore, given that suicidal behaviour is a complex process resulting from an intricate interplay of biological, psychological, environmental and situational factors (Wasserman et al. 2012), there is a need for an element of flexibility to adapt the therapy to fulfil the individual needs of the client.

The underlying tenet of the proposed model is that the psychological turmoil (Shneidman, 1993) can be mediated by protective factors such as coping strategies, healthy lifestyles, physical exercise, personal value, self-confidence, and communication skills (Wasserman 2001). The goal of therapy is to resolve the client’s suicidal crisis and improve their outlook for the future by enhancing protective factors that enable them to overcome current and future crises.

### Existing crisis intervention methods

Existing crisis intervention models provide something of a dichotomy. As Thomas and Leitner (2005) report current intervention models and standard protocol are rooted in the theoretical framework established by the Los Angeles Suicide Prevention Center in 1958. Consequently, while the number of stages varies from model to model (e.g., two stages (Berman and Jobes 1997), three stages (Stanley et al. 2009), or seven stages (Roberts 1991), (Granello 2010)) there is a considerable degree of consensus on the structure of the intervention: a pre-therapy; therapy and consolidation; and follow up. However, while this framework has been clearly established there has been less clarity around precisely how these should be enacted (Thomas and Leitner 2005), resulting in a proliferation of differing approaches. This difficulty has been further confounded by a lack of empirical evidence.

Thomas et al. (2009) reported that most suicidal patients are treated with unproven therapies, a sentiment echoed by Jobes (2013) who commented on the ‘remarkably un-evolved and surprisingly limited’ knowledge of effective intervention models and concluded that many approaches used have ‘little to no empirical support’ (p.127). Models that have been forwarded face the same difficulty of the original structures and protocols – a clear structure but lack of detail that makes replication impossible. For example, Sanchez’s (2001) model incorporates both risk and protective factors that would facilitate risk assessment and the development of therapy interventions, but provided no details of how therapy should then be enacted.

Consequently the search for a flexible, yet clearly defined, evidence-based intervention therapy model with provision for both risk and protective factors proved to be unsuccessful. Instead, a new therapy model is proposed below.

### Developing the Pieta House Suicide Intervention Model

**Figure 1:**
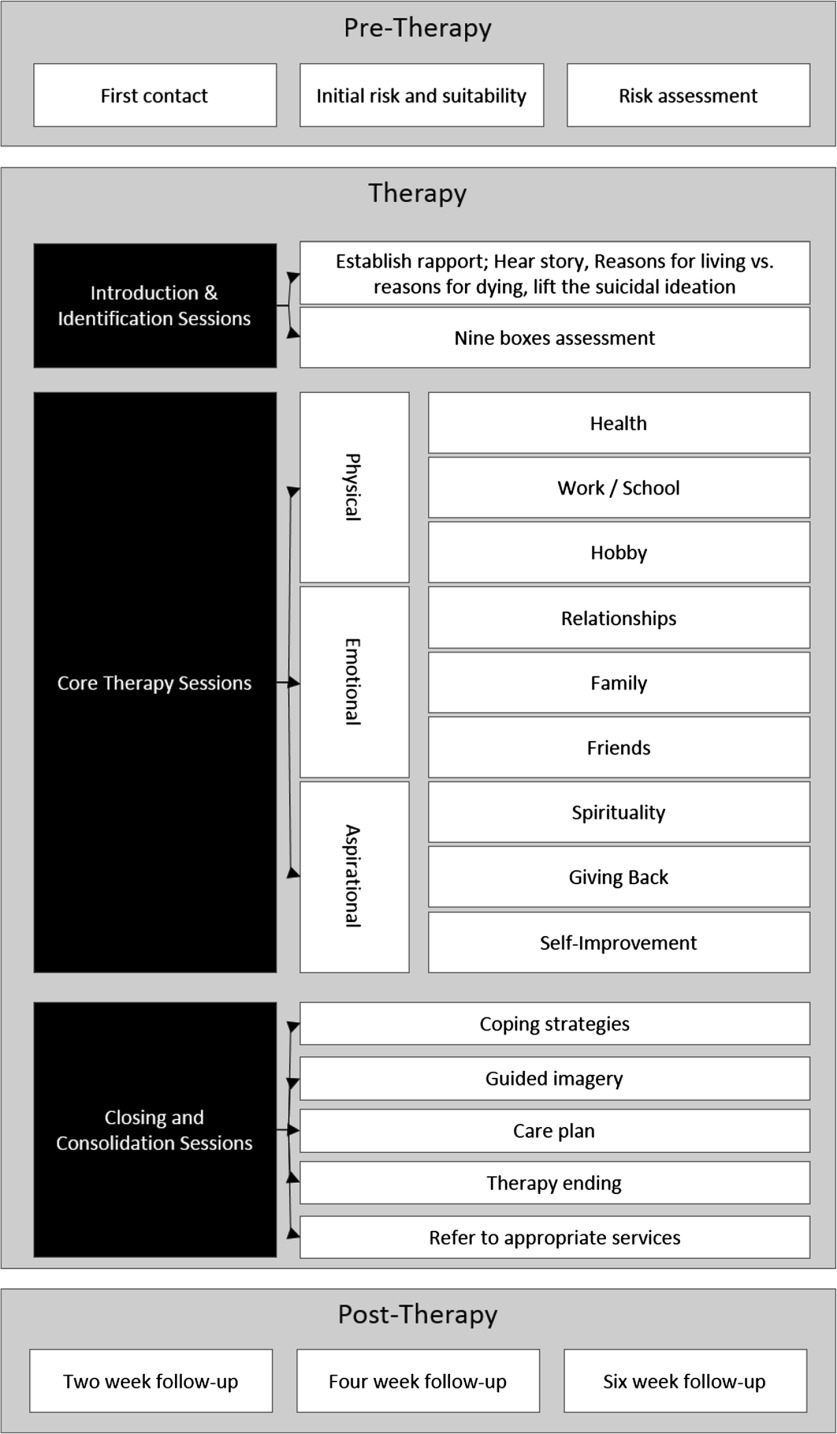
The Pieta House Suicide Intervention Model (PH-SIM).

The proposed Pieta House Suicide Intervention Model (PH-SIM) is presented in Figure 1. In line with existing intervention models it has risk assessment (Pre-Therapy), therapy and consolidation (Therapy), and follow-up support (Follow-up) stages.

1. Pre-therapy stage

While participation is encouraged for all there are some for whom therapy at Pieta House would be unsuitable due to their inability to engage in one-on-one dialectical sessions with a therapist. Clients are unsuitable for therapy if they suffer from severe mental ill-health, a severe intellectual disability or behavioural disorder, or an active alcohol or substance addiction. A comprehensive risk assessment is administered for new clients during an initial meeting where, based on Granello’s (2010) suggestion of rearranging the traditional seating convention, clients sit on a comfortable chair while the therapist sits on a lower chair to emphasise the lack of hierarchy.

2. Therapy stage

The core therapy sessions at Pieta House involve developing the client’s protective factors in diverse areas of their lives. This uses an adaptation of Jeffers’ (1988) Nine Boxes to visually illustrate areas in which they have sufficiently developed, underdeveloped, or no protective factors. The nine areas targeted by the model address physical, social, and aspirational needs, three components important for human contentment (Snyder and Lopez 2002). The areas addressed by each stage of therapy is outlined below.

Sessions 1 and 2: The opening sessions are dedicated to hearing the client’s story and establishing a rapport. This follows a ‘listen, understand, validate’ approach (Echterling et al. 2005) to establish a genuine, caring, and non-judgmental therapeutic environment where therapist and client work together to explore issues and solutions (Jobes 2006). After initial discussions exploring reasons for living and for dying the Nine Boxes are introduced. This allows the therapist to guide the client through a collaborative process of identifying the areas in their life where they have adequate protective factors. Any area that is sufficiently developed need not be addressed in the course of therapy. In this way the therapist and client co-create a bespoke therapy programme to specifically develop protective factors where they’re needed most.

The environment plays an important part in the therapy process. The therapy centre is designed to resemble a comfortable family home rather than a formal clinical setting, with therapists receiving specific guidelines on all aspects of the therapy, such as the physical distance between the therapist and client (18 inches, the nexus point of personal space and personal distance (Thompson and Hickey 2005), and tone of voice (slow, calm, controlled, and using short sentences and ‘downspeak’ (Bradford 1997)).

Sessions 3 to 13: After the immediate suicidal crisis has been addressed and the areas for development identified, the next priority is to develop skills in these areas to promote recovery and safeguard against future crises (Stanley et al. 2009). The same approach is adopted for each of the three components, and involves the use of CBT, DBT, and problem-solving strategies. Approved CBT activities aim to change patterns of dysfunctional thinking and improve mood and behaviour (Furlong and Oei 2002); DBT activities include mindfulness, validation, targeting and chain analyses as mechanisms of change (Cutcliffe and Santos 2012) to aid in emotional regulation; and problem-solving activities aim to help identify effective means of coping with problems of everyday living (Cully and Teten 2008). In each case concrete, solution-focused, achievable plans (Chiles and Strosahl 2005) are jointly developed.

The three components and their associated protective factors are briefly discussed in turn.

- Physical needs
  Increased physical activity has been associated with improved cognitive functioning (Etnier et al. 2006), better quality of life (Brown et al. 2004), and decreased suicidal ideation (Brown et al. 2007). The ‘physical needs’ component encapsulates three factors: health, hobby, and employment. In the first of these a physical activity plan is devised and implemented in conjunction with friends and family members (Encrenaz et al. 2012). The ‘hobby’ factor aims to stimulate interest in previously enjoyable pursuits as a means of engaging in positive and affirming activities, and consolidating internal coping strategies (Stanley et al. 2008). The link between suicidal ideation and unemployment/employment difficulties is well established (Corcoran and Arensman 2011; Kposowa 2001; Platt and Hawton 2000; Wong et al. 2008) and the ‘employment’ factor involves assisting the client to positively appraise current employment issues or addressing concerns of unemployment.
- Emotional needs
  The client’s emotional needs are explored through three factors: family; friends; and relationships. Research (Durkheim 1952; Helliwell 2007; Mignone and O’Neil 2005) has provided an indication of the protection afforded by the social support afforded by family and community, and the risk factor of isolation and absence of a significant relationship (Granello 2010). In the eventuality that a family connection or existing friendship cannot be identified, a relationship with any significant other is explored.
- Aspirational needs
  Clients are encouraged to explore at least one of the three factors of this component (spirituality, altruism, and self-improvement) with the aim of developing a sense of fulfilment, belonging, and worth. The term ‘spiritualty’ is used very loosely and refers to the beliefs or support structures that have been shown to provide a protective influence (Gearing and Lizardi 2009; Hilton et al. 2002; Koenig et al. 2001; Linehan et al. 1983; Szanto et al. 2003), even across denominational divides (Dervic et al. 2004). The altruism factor encourages clients to consider how they can ‘give something back’ by reinvesting in a community of their choice. This directly relates to the concept of social capital which has been identified as having a protective effect on suicidal ideation (Patel 2010). In relation to self-improvement, the client is encouraged to identify an area in which they would like to enhance existing, or undertake new, skills and abilities. This develops self-esteem (Macdonald 1994), resilience and confidence, and provides a rationale for living (Granello 2010).
  Sessions 13 to 15: Consolidation of the coping strategies developed is established through the use of guided-imagery to explore responses to potential suicide-related crises and behaviour (Henriques et al. 2003), and follows the five-step process outlined by Stanley et al. (Stanley et al. 2009). Clients are also warned of the potential for recurrence of suicidal thinking and are encouraged to adhere to the care plan developed throughout therapy that provides the skills required confront future crises.

3. Follow-up

In line with existing suicide intervention models (Granello 2010; Huisman et al. 2010; Roberts 1991; Stanley et al. 2009) and the recommendation of previous research (Macdonald et al. 2009) the PH-SIM concludes with a follow-up period with the client. The first follow-up contact is a text message two weeks after therapy has concluded to serve as a brief reminder that the service is available when required. Four weeks after therapy has concluded the client receives a letter and information on local support services they may find useful to deal with more specific stressors (e.g., relationship or financial issues). The final contact occurs six weeks after therapy has concluded and is a telephone call to check on the client’s progress and suicidal ideation. As advised by Mann (2002) this enquires about their current depression, hopelessness, and suicidal ideation. If the therapist is satisfied with the client’s progress the therapy is officially closed.

### Aims

While the proposed model is established on existing intervention structures, fulfils the therapeutic requirements of the organisation, and permits for adaptation to meet the client’s needs, it is necessary to evaluate its ability to decrease suicidal ideation and increase the desire to live. The aim of this research, then, is to assess the impact of engaging in the proposed therapy model on clients’ negative and positive suicidal outlook. This will be achieved by comparing levels of suicidal ideation, depression, and self-esteem of clients in suicidal crisis before any therapy has begun, with levels recorded in the month following the completion of their therapy. It is hypothesized that clients will have a decreased negative outlook (i.e. lower levels of depression and negative suicidal ideation) and more positive outlook (i.e. greater self-esteem and reasons for living) after engaging in therapy using the PH-SIM.

## Methods

### Experimental design

This study employed a quasi-experimental, pre-test post-test design without a control group.

### Sample

A total of 432 of the 664 invited to participate in the pre-therapy stage did so (65.1%), of which 44.4% were male and 55.6% were female. Post-therapy, 147 clients (50.3%) continued to participate (50.3% males and 49.7% females). This figure exceeds the required 44 clients the G*Power 3 programme (Faul et al. 2009) calculated as necessary for a MANOVA to detect large effects (.40) with 95% power at the .05 significance level. The age range was from 18 to 74 years old, with a mean of 38.1 years (sd = 13.7).

### Research tool

The questionnaire was designed to be as short as possible due to the vulnerable condition of the clients, particularly pre-therapy. Information on the scales used is presented below.

#### Self-esteem

Self-esteem was measured by Robins et al. (2001) single-item indicator (“I have high self-esteem”) which is rated on a five-point scale and has been shown to have a very high convergent validity with the Rosenberg Self-Esteem Scale (Rosenberg 1965).

#### Depression

The Patient Health Questionnaire (PHQ-9) is a nine-item scale for assessing the severity of depression (Kroenke et al. 2001). It has well-established reliability and validity when administered face-to-face or over the telephone (Pinto-Meza et al. 2005). The scale asks about the frequency of activities over the past two weeks relating to eating, sleeping, energy and motivation levels, and responses range from zero (‘not at all’) to three (‘nearly every day’).

#### Positive and negative suicidal ideation

The Positive and Negative Suicide Ideation Inventory (PANSI) (Osman et al. 1998) assesses the frequency of factors that increase the client’s desire to die (their Negative Suicidal Ideation) and those that serve to protect the client by increasing coping, resilience, or social support to decrease suicidal ideation and enhance their desire to live (their Positive Suicidal Ideation). To keep the questionnaire as short as possible four items were selected from the positive scale (items 2, 12, 13, and 14) and four from the negative scale (items 1, 3, 5, and 11) based on the strength of the factor loadings on the confirmatory factor analysis conducted by Osman et al. (Osman et al. 2002).

Positive outlook is measured by self-esteem and positive suicidal ideation, and negative outlook by depression and negative suicidal ideation.

### Procedure

The pre-therapy questionnaire was administered by the therapist at the initial assessment before any therapy had commenced. Clients were invited to participate and provided with an information sheet. After participating clients provided written informed consent for participation in the study, questions were read aloud by the therapist and responses recorded on the questionnaire. After their therapy had been completed participants were called by independent researchers within a month and the same questions administered via telephone. This enabled clients’ pre- and post-therapy responses to be matched. The study received ethical approval from the Research Ethics Committee at the Adelaide & Meath Hospital, Incorporating the National Children’s Hospital in Dublin.

## Results

Repeated measures MANOVAs were conducted to establish whether therapy using the PH-SIM had a significant effect on clients’ negative and positive outlook.

### Negative outlook

#### Depression

Analysis revealed a statistically significant overall effect suggesting that therapy was a significant predictor of depression (F (1.63, 99.5) = 15.34, p < .01, ηp.2 = .20). Follow-up paired-samples t-tests between pre-therapy and post-therapy levels revealed a significant difference (see Table 1), with statistically lower scores after therapy.

**Table 1:** **Paired-samples t-tests for pre- and post-treatment scores**

**Measure**	**Pre-treatment**		**Post-treatment**		**T values and significance**
	***Mean***	***SD***	***Mean***	***SD***	
Depression	18.58	5.77	10.87	7.47	t (92) = 9.07, p < .001
Neg. suicidal ideation	13.04	4.22	7.77	4.82	t (81) = 9.58, p < .001
Self-esteem	1.76	1.07	2.79	1.08	t (91) = −6.80, p < .001
Pos. suicidal ideation	9.48	3.69	13.76	3.66	t (82) = −7.62, p < .001

#### Negative suicide ideation

The significant effect for therapy (F (2, 53) = 38.7, p < .01, ηp.2 = .59) suggests that this was a significant predictor of negative suicidal ideation. Follow-up analyses of the pre- and post- therapy scores (see Table 1) reveals significantly lower levels of negative suicidal ideation after therapy had finished.

### Positive outlook

#### Self-esteem

Results of a within-subjects repeated-measures MANOVA revealed a statistically significant overall effect for self-esteem (F (2, 62) = 27.58, p < .01, ηp.2 = .47), with statistically significant higher scores noted post-therapy (see Table 1).

#### Positive suicide ideation

The statistically significant overall effect (F (2, 55) = 26.0, p < .01, ηp.2 = .49) suggests that engaging in therapy was a significant predictor of positive outlook. The mean difference on the follow-up t-tests between pre- and post-therapy levels indicated statistically significant higher levels of positive outlook after therapy had finished.

The results show that clients’ negative outlook (as measured by depression and negative suicidal ideation) had significantly decreased, while positive outlook (self-esteem and positive suicidal ideation) had significantly increased after therapy with the PH-SIM had been completed.

## Discussion

There is no consensus on what makes suicide crisis intervention therapy effective (Thomas et al. 2009). While most intervention models adhere to the same generalised structure (pre-therapy, therapy, post-therapy) the lack of detail provided on the content, progression, or protocol has resulted in the development and use of myriad models (Thomas and Leitner 2005), most of which have little or no empirical basis (Jobes 2013).

The PH-SIM is an intervention model that, unlike many of its predecessors, provides sufficient information to enable a therapist to replicate the therapy process. It was developed due to the inability to find an evidence-based intervention model that was based primarily on the development of multiple protective factors. The proposed model was designed to increase the client’s positive outlook (their reason for living) while decreasing their negative outlook (their reasons for dying) by developing new, or reinforcing existing, protective factors in nine specified areas of their life.

This research aimed to establish the impact of the proposed therapeutic model on clients’ outlook by comparing levels positive and negative outlook before and after therapy. The significant effects and the decreased negative and increased positive outlook following therapy provide some support for the PH-SIM in fulfilling its role. These results are reported cautiously and with acknowledgement of the absence of a randomised control group, a small sample size, and the possibility of regression to the mean.

Further research will explore the longitudinal impact of therapy using the PH-SIM on client outlook, the means by which the therapeutic process affects risk and protective factors, and the linkages between specific protective factors and levels of suicidal ideation.

### Limitations

The study had several limitations that may affect the generalizability of the findings. Firstly, the study employed no control group as this would involve denying some clients the therapy programme provided by Pieta House which runs contrary to the principle of beneficence as outlined in the Belmont Report (National Commission for the Protection of Human Subjects of Biomedical and Behavioral Research 1979). The repeated-measures design was used instead, in an attempt to reduce error variance (Ellis 1999) and provide control over threats to internal validity (Huck and McLean 1975). Secondly, there were a number of factors that increased the proportion of missing values. Since the study was designed to determine the impact of completing therapy using the proposed model, only those that had fully completed their therapy programme were included in the sample. Future research should explore the impact of therapy on those who did not complete their programme. The main reason cited by participants for post-assessment non-participation was that they had progressed beyond their suicidal crisis and were reluctant to revisit that aspect of their life. This smaller post-therapy sample has an impact on the generalizability of the findings. The issue of missing values in future studies could be addressed by providing clients with better information on the research and its follow-up component pre-therapy, by maintaining a degree of contact with clients in the period between ending therapy and questionnaire administration, through closer liaison with the organisation’s service-user panel, or by the use of a multiple imputation strategy.

## Conclusions

This research aimed to propose a detailed suicide intervention model, and to assess the impact of therapy using this model on clients’ negative and positive suicidal outlook. The main function of the model was to resolve current and future crises by developing protective factors in multiple areas of their life. Comparison of data before and after therapy suggests that clients who engaged in therapy had significantly lower levels of negative outlook and significantly higher levels of positive outlook. While additional research is required to establish the exact role of the model in achieving these results, this provides some initial support for the proposed suicide intervention model.

## References

[CR1] Berman A, Jobes D (1997). Adolescent suicide assessment and intervention.

[CR2] Bradford B (1997). Upspeak in British English. English Today.

[CR3] Brown DW, Brown DR, Heath GW, Balluz L, Giles WH, Ford ES, Mokdad AH (2004). Associations between physical activity dose and health-related quality of life. Medicine and Science in Sports and Exercise.

[CR4] Brown DR, Galuska DA, Zhang J, Eaton DK, Fulton JE, Lowry R, Maynard LM (2007). Physical activity, sport participation, and suicidal behavior: U.S. High School students. Medicine and Science in Sports and Exercise2.

[CR5] Chiles JA, Strosahl KD (2005). Clinical manual for assessment and treatment of suicidal patients.

[CR6] Corcoran P, Arensman E (2011). Suicide and employment status during Ireland’s Celtic Tiger economy. European Journal of Public Health.

[CR7] Cully JA, Teten AL (2008). A Therapist’s Guide to Brief Cognitive Behavioral Therapy.

[CR8] Cutcliffe J, Santos J (2012). Suicide and self-harm: an evidence-informed approach.

[CR9] Dervic K, Oquendo MA, Grunebaum MF, Ellis S, Burke AK, Mann JJ (2004). Religious affiliation and suicide attempt. The American Journal of Psychiatry.

[CR10] Durkheim E (1952). Suicide: A study in sociology.

[CR11] Echterling LG, Presbury J, McKee JE (2005). Crisis intervention: Promoting resilience and resolution in troubled times.

[CR12] Ellis ME (1999). Repeated measures designs. The Counselling Psychologist.

[CR13] Encrenaz G, Kovess-Masféty V, Gilbert F, Galéra C, Lagarde E, Mishara B, Messiah A (2012). Lifetime risk of suicidal behaviors and communication to a health professional about suicidal ideation. Results from a large survey of the French adult population. Crisis: The Journal of Crisis Intervention and Suicide Prevention.

[CR14] Etnier JL, Nowell PM, Landers DM, Sibley BA (2006). A meta-regression to examine the relationship between aerobic fitness and cognitive performance. Brain Research Reviews.

[CR15] Faul F, Erdfelder E, Buchner A, Lang AG (2009). Statistical power analyses using G*Power 3.1: Tests for correlation and regression analyses. Behavior Research Methods.

[CR16] Furlong M, Oei TP (2002). Changes to automatic thoughts and dysfunctional attitudes in group CBT for depression. Behavioural and Cognitive Psychotherapy.

[CR17] Gearing RE, Lizardi D (2009). Religion and suicide. Journal of Religion and Health.

[CR18] Granello D (2010). A suicide crisis intervention model with 25 practical strategies for implementation. Journal of Mental Health Counseling.

[CR19] Helliwell J (2007). Well-being and social capital: does suicide pose a puzzle. Social Indicators Research.

[CR20] Henriques GR, Beck AT, Brown GK (2003). Cognitive therapy for adolescent and young adult suicide attempters. American Behavioral Scientist.

[CR21] Hilton SC, Fellingham GW, Lyon JL (2002). Suicide rates and religious commitment in young adult males in Utah. Journal of Epidemiology and Community Health.

[CR22] Huck SW, McLean RA (1975). Using a Repeated Measures ANOVA to Analyze the Data from a Pretest-Posttest Design: A Potentially Confusing Task. Psychological Bulletin.

[CR23] Huisman A, Pirkis J, Robinson J (2010). Intervention studies in suicide prevention research. Crisis: The Journal of Crisis Intervention and Suicide Prevention.

[CR24] Jeffers S (1988). Feel the fear and do it anyway.

[CR25] Jobes DA (2006). Managing suicidal risk: A collaborative approach.

[CR26] Jobes DA (2013). Reflections on suicide among soldiers. Psychiatry.

[CR27] Koenig HG, McCullough ME, Larson DB (2001). Handbook of Religion and Health.

[CR28] Kposowa AJ (2001). Unemployment and suicide: a cohort analysis of social factors predicting suicide in the US National Longitudinal Mortality Study. Psychological Medicine.

[CR29] Kroenke K, Spitzer RL, Williams JB (2001). The PHQ-9: validity of a brief depression severity measure. Journal of General Internal Medicine.

[CR30] Linehan MM, Stoff DM, Mann JJ (1997). Behavioral treatments of suicidal behaviors: Definitional obfuscation and treatment outcomes. Neurobiology of suicide: From the bench to the clinic.

[CR31] Linehan MM (2008). Suicide intervention research: a field in desperate need of development. Suicide and Life-Threatening Behavior.

[CR32] Linehan MM, Goodstein JL, Nielsen SL, Chiles JA (1983). Reasons for staying alive when you are thinking of killing yourself: The reasons for living inventory. Journal of Consulting and Clinical Psychology.

[CR33] Macdonald G, Trent D, Reed C (1994). Self-esteem and the promotion of mental health. Promotion of Mental Health.

[CR34] Macdonald L, Pelling N, Granello DH (2009). Suicide: a biopsychosocial approach. Psychotherapy in Australia.

[CR35] Mann JJ (2002). A current perspective of suicide and attempted suicide. Annals of Internal Medicine.

[CR36] Mignone J, O’Neil J (2005). Social capital and youth suicide risk factors in First Nations Communities. Canadian Journal of Public Health.

[CR37] National Commission for the Protection of Human Subjects of Biomedical and Behavioral Research (1979). The Belmont Report: Ethical principles and guidelines for the protection of human subjects of research. OPRR Reports.

[CR38] National Office for Suicide Prevention (2005). Reach out: National strategy for action on suicide prevention 2005–2014.

[CR39] Office of the Surgeon General (US); National Action Alliance for Suicide Prevention (US). (2012). 2012 National Strategy for Suicide Prevention: Goals and Objectives for Action: A Report of the U.S. Surgeon General and of the National Action Alliance for Suicide. Washington (DC). Retrieved from http://www.ncbi.nlm.nih.gov/books/NBK109917/.

[CR40] Osman A, Gutierrez PM, Kopper BA, Barrios FX, Chiros CE (1998). The positive and negative suicide ideation inventory: development and validation. Psychological Reports.

[CR41] Osman A, Barrios F, Gutierrez PM, Wrangham JJ, Kopper BA, Truelove RS, Linden SC (2002). The Positive and Negative Suicide Ideation (PANSI) inventory: psychometric evaluation with adolescent psychiatric inpatient samples. Journal of Personality Assessment.

[CR42] Patel V (2010). Building social capital and improving mental health care to prevent suicide. International Journal of Epidemiology.

[CR43] Pinto-Meza A, Serrano-Blanco A, Penarrubia MT, Blanco E, Haro JM (2005). Assessing depression in primary care with the PHQ-9: can it be carried out over the telephone?. Journal of General Internal Medicine.

[CR44] Platt S, Hawton K, Hawton K, Van Heeringen K (2000). Suicidal behaviour and the labour market. The international handbook of suicide and attempted suicide.

[CR45] Ramsay R (2004). New developments in suicide intervention training. Suicidologi.

[CR46] Roberts AR, Roberts AR (1991). Conceptualizing crisis theory and the crisis intervention model. Contemporary perspectives on crisis intervention and prevention.

[CR47] Roberts AR (2000). Crisis Intervention Handbook: Assessment, treatment, and research.

[CR48] Robins RW, Hendin HM, Trzesniewski KH (2001). Measuring global self-esteem: construct validation of a single-item measure and the rosenberg self-esteem scale. Personality and Social Psychology Bulletin.

[CR49] Rosenberg M (1965). Society and the adolescent self-image.

[CR50] Sanchez H (2001). Risk factor model for suicide assessment and intervention. Professional Psychology and Practice.

[CR51] Shneidman E (1985). Definition of suicide.

[CR52] Shneidman ES (1993). Suicide as psychache. The Journal of Nervous and Mental Disease.

[CR53] Snyder CR, Lopez SJ (2002). Handbook of positive psychology.

[CR54] Stanley B, Brown GK, Karlin B, Kemp JE, VonBergen HA (2008). Safety plan treatment manual to reduce suicide risk: veteran version.

[CR55] Stanley B, Brown G, Brent D, Wells K, Poling K, Curry J, Hughes J (2009). Cognitive behavioral therapy for suicide prevention (CBT-SP): Treatment model, feasibility, and acceptability. American Academy of Child and Adolescent Psychiatry.

[CR56] Szanto K, Mulsant BH, Houck P, Dew MA, Reynolds CF (2003). Occurrence and course of suicidality during short-term treatment of late-life depression. Archives of General Psychiatry.

[CR57] Thomas JC, Leitner LM (2005). Styles of suicide intervention: professionals’ responses and clients’ preferences. The Humanistic Psychologist.

[CR58] Thomas EE, Allen JG, Harrell Woodson B, Frueh C, Jobes DA (2009). Implementing an evidence-based approach to working with suicidal inpatients. Bulletin of the Menninger Clinic.

[CR59] Thompson WE, Hickey JV (2005). Society in Focus.

[CR60] Wasserman D, Wasserman D (2001). A stress-vulnerability model and the development of the suicidal process. Suicide: An unnecessary death.

[CR61] Wasserman D, Rihmer Z, Rujescu D, Sarchiapone M, Sokolowski M, Titelman D, Carli V (2012). The European Psychiatric Association (EPA) guidance on suicide treatment and prevention. European Psychiatry: The Journal of the Association of European Psychiatrists.

[CR62] Wong PW, Chan WS, Chen EY, Chan SS, Law YW, Yip PS (2008). Suicide among adults aged 30–49: a psychological autopsy study in Hong Kong. BMC Public Health.

